# Investigation of *methylenetetrahydrofolate reductase* tagging polymorphisms with colorectal cancer in Chinese Han population

**DOI:** 10.18632/oncotarget.18845

**Published:** 2017-06-29

**Authors:** Sheng Zhang, Shuchen Chen, Yu Chen, Mingqiang Kang, Chao Liu, Hao Qiu, Yafeng Wang, Weifeng Tang

**Affiliations:** ^1^ Department of General Surgery, Changzhou No. 3 People's Hospital, Changzhou, Jiangsu Province, China; ^2^ Department of Thoracic Surgery, Affiliated Union Hospital of Fujian Medical University, Fuzhou, Fujian Province, China; ^3^ Department of Medical Oncology, Fujian Provincial Cancer Hospital, Fujian Medical University Cancer Hospital, Fuzhou, Fujian Province, China; ^4^ Fujian Provincial Key Laboratory of Translational Cancer Medicine, Fuzhou, Fujian Province, China; ^5^ Department of Cardiothoracic Surgery, Affiliated People's Hospital of Jiangsu University, Zhenjiang, Jiangsu Province, China; ^6^ Department of Immunology, School of Medicine, Jiangsu University, Zhenjiang, Jiangsu Province, China; ^7^ Department of Cardiology, The People's Hospital of Xishuangbanna Dai Autonomous Prefecture, Jinghong, Yunnan Province, China

**Keywords:** polymorphism, MTHFR, colorectal cancer, tagging, susceptibility

## Abstract

The aim of this case-control study was to assess the relationship between the tagging polymorphisms in *methylenetetrahydrofolate reductase* (*MTHFR*) gene and the susceptibility to colorectal cancer (CRC) in a Chinese Han population. A custom-by-design 48-Plex SNPscan Kit was used to determine the genotypes of *MTHFR* rs3753584 T>C, rs9651118 T>C, rs1801133 G>A, rs4846048 A>G and rs4845882 G>A polymorphisms in 387 CRC patients and 1,536 non-cancer controls. The results revealed that *MTHFR* rs1801133 G>A polymorphism was associated with a decreased risk of overall CRC. While *MTHFR* rs4845882 G>A polymorphism conferred an increased risk to overall CRC. In a stratified analysis by CRC region, we found *MTHFR* rs3753584 T>C and rs9651118 T>C polymorphisms were associated with the increased risk of colon cancer. In addition, a significantly increased risk of rectum cancer associated with *MTHFR* rs3753584 T>C polymorphism was overt. However, *MTHFR* rs1801133 G>A polymorphism conferred a decreased risk to colon cancer. In conclusion, findings of the present study reveal that the tagging polymorphisms in *MTHFR* gene (rs3753584 T>C, rs9651118 T>C and rs4845882 G>A) are associated with the increased risk of CRC. However, *MTHFR* rs1801133 G>A polymorphism confers a decreased risk to CRC. Additional studies with larger sample size are needed to confirm these findings.

## INTRODUCTION

Colorectal cancer (CRC) is the fourth most frequent type of malignancy among females and the fifth most frequent type among males in China, accounting for 160,600 and 215,700 cases in 2015, respectively [1]. The CRC morbidity is shooting up in developing countries including China [1, 2]; nevertheless, CRC etiology remains unknown. Risk factors, such as advanced age, family history of CRC, benign adenomatous polyp, inflammatory bowel disease, drinking, smoking, being physically inactive, low intake of fruits and vegetables and high intake of dietary fat, may play important roles in the occurrence and the development of CRC [3–9]. Accumulating evidences indicated that besides environmental factors and individual lifestyle, some genetic factors might be related to CRC etiology.

Several studies have found association between circulating or dietary folate level and the risk of CRC [10–12]. A long induction period deserves close attention to the study of CRC because the course from normal cells of the rectum and colon, to microadenomas, to macroadenomas, and eventually to adenocarcinomas, experiences over a long duration, approximately 30-40 years [13]. The presence of the long induction period between reduced risk of CRC and adequate folate status is evident in both epidemiologic and molecular mechanistic studies [14]. The lower folate levels lead to an increasing rate of DNA hypomethylation and uracil misincorporation even in non-neoplastic, normal-appearing tissue of cases [14]. Folate is reduced to tetrahydrofolate (THF) to participate in one-carbon metabolism [15]. Folate metabolism is accommodated by several enzymes. Methylenetetrahydrofolate reductase (MTHFR) is the key enzyme [16–18]. MTHFR is involved in DNA methylation, repair and synthesis [19]. DNA strand break and repair, and impaired DNA methylation have been associated with folate deficiency and CRC.

*MTHFR* gene locates on 1p36.22. The gene encodes a 74.6-kDa protein containing 656 amino acids. MTHFR is also linked to purine synthesis; therefore, plays a vital role in DNA synthesis by the provision of essential nucleotides [18]. Single nucleotide polymorphisms (SNPs) of *MTHFR* are considered as a potential biomarker which may influence CRC risk. Therefore, we carried out this case-control study in a Chinese Han population to determine whether *MTHFR* tagging SNPs (rs3753584 T>C, rs9651118 T>C, rs1801133 G>A, rs4846048 A>G and rs4845882 G>A) were associated with the risk of CRC.

## RESULTS

### Demographic characteristics

The frequency distributions of age, sex, smoking status and drinking habit information for the 387 patients with CRC (mean ± SD age, 60.21 ± 12.48 years) and 1,536 controls (mean ± SD age, 60.82± 8.85 years) are shown in Table 1. The age and sex of the CRC patients and controls were well-matched. The differences of smoking status and drinking habit between CRC and non-cancer controls were not statistically significant (*P* ≥ 0.05) (Table 1). Tumor grade was defined as high (well differentiated), medium (moderately differentiated); and low (poorly differentiated). Two experienced doctors independently assessed disease stage according to the AJCC criteria (2010). Finally, 196 cases with stage I/II and 191 with stage III/IV were included. Among the CRC patients, 218 were rectum cancer and 169 were colon cancer. And the primary information for *MTHFR* polymorphisms is summarized in Table 2.

**Table 1:** Distribution of selected demographic variables and risk factors in colorectal cancer cases and controls

Variable	Cases (n=387)		Controls (n=1,536)		*P*^a^
	n	%	n	%	
Age (years)	60.21 (±12.48)		60.82 (±8.85)		0.272
Age (years)					0.502
< 61	186	48.06	709	46.16	
≥ 61	201	51.94	827	53.84	
Sex					0.213
Male	236	60.98	989	64.39	
Female	151	39.02	547	35.61	
Smoking status					0.505
Never	270	69.77	1098	71.48	
Ever	117	30.23	438	28.52	
Alcohol use					0.058
Never	335	78.55	1381	89.91	
Ever	52	21.45	155	10.09	
Site of tumor					
Colon cancer	169	43.67			
Rectum cancer	218	56.33			
Degree of differentiation^b^					
Low	56	16.28			
Medium	261	75.87			
High	27	7.85			
Lymph node status					
Positive	177	45.74			
Negative	210	54.26			
TMN stage					
I+II	196	50.65			
III+IV	191	49.35			

^a^Two-sided *χ*^2^ test and Student t test.

**Table 2:** Primary information for *MTHFR* polymorphisms (rs3753584 T>C, rs9651118 T>C, rs1801133 G>A, rs4846048 A>G and rs4845882 G>A)

Genotyped SNPs	rs3753584 T>C	rs9651118 T>C	rs1801133 G>A	rs4846048 A>G	rs4845882 G>A
Chromosome	1	1	1	1	1
Function	NearGene-5	Intron	Missense	Intron	Intron
Chr Pos (Genome Build 36.3)	11787173	11784801	11778965	11768839	11765754
MAF^a^ for Chinese in database	0.093	0.382	0.439	0.105	0.198
MAF in our controls (n = 1,536)	0.109	0.379	0.356	0.096	0.209
*P* value for HWE^b^ test in our controls	0.164	0.134	0.747	0.082	0.733
Genotyping method	SNPscan	SNPscan	SNPscan	SNPscan	SNPscan
% Genotyping value	99.64%	99.64%	99.64%	99.64%	99.64%

^a^MAF: minor allele frequency;

^b^HWE: Hardy–Weinberg equilibrium.

### Association of MTHFR rs3753584 T>C, rs9651118 T>C, rs1801133 G>A, rs4846048 A>G and rs4845882 G>A polymorphisms with CRC patients

The genotypes of *MTHFR* rs3753584 T>C, rs9651118 T>C, rs1801133 G>A, rs4846048 A>G and rs4845882 G>A polymorphisms in CRC patients and non-cancer controls are summarized in Table 3. The observed genotype frequencies for the five *MTHFR* tagging SNPs in control group were all in HWE (*P* = 0.134, 0.082, 0.733, 0.164 and 0.747 for *MTHFR* rs9651118 T>C, rs4846048 A>G, rs4845882 G>A, rs3753584 T>C and rs1801133 G>A, respectively), which suggested good homogeneity within the present study participants. Four CRC patients (1.03%) and three controls (0.19%) could not be genotyped for poor DNA quantity and/or quality. Overall, we found no statistically significant difference in genotype distribution of *MTHFR* rs3753584 T>C, rs9651118 T>C and rs4846048 A>G polymorphisms among CRC patients and controls (Table 4).

**Table 3:** The frequencies of *MTHFR* rs3753584 T>C, rs9651118 T>C, rs1801133 G>A, rs4846048 A>G and rs4845882 G>A polymorphisms in colorectal cancer patients and controls

Genotype	Colorectal cancer (n=387)		Colon cancer	(n=169)	Rectum cancer (n=218)		Controls	(n=1,536)
	n	%	n	%	n	%	n	%
*MTHFR* rs3753584 T>C								
TT	287	74.93	131	78.44	156	72.22	1211	79.00
TC	89	23.24	31	18.56	58	26.85	309	20.16
CC	7	1.83	5	2.99	2	0.93	13	0.85
CT+CC	96	25.07	36	21.56	60	27.78	322	21.00
TT+CT	376	98.17	162	97.01	214	99.07	1,520	99.15
CC	7	1.83	5	2.99	2	0.93	13	0.85
C allele	103	13.45	41	12.28	62	14.35	335	10.93
*MTHFR* rs9651118 T>C								
TT	131	34.20	56	33.53	75	34.72	578	37.70
TC	188	49.09	79	47.31	109	50.46	749	48.86
CC	64	16.71	32	19.16	32	14.81	206	13.44
TC+CC	252	65.80	111	66.47	141	65.28	955	62.30
TT+TC	319	83.29	135	80.84	184	85.19	1,327	86.56
CC	64	16.71	32	19.16	32	14.81	206	13.44
C allele	316	41.25	143	42.81	173	40.05	1,161	37.87
*MTHFR* rs1801133 G>A								
GG	177	46.21	82	49.10	95	43.98	639	41.68
GA	175	45.69	73	43.71	102	47.22	697	45.47
AA	31	8.09	12	7.19	19	8.80	197	12.85
GA + AA	206	53.79	85	50.90	121	56.02	894	58.32
GG+ GA	352	91.91	155	92.81	197	91.20	1,336	87.15
AA	31	8.09	12	7.19	19	8.80	197	12.85
A allele	237	30.94	97	29.04	140	32.41	1,091	35.58
*MTHFR* rs4846048 A>G								
AA	308	80.42	136	81.44	172	79.63	1259	82.13
AG	70	18.28	30	17.96	40	18.52	254	16.57
GG	5	1.31	1	0.60	4	1.85	20	1.30
AG+GG	75	19.58	31	18.56	44	20.37	274	17.87
AA+AG	378	98.69	166	99.40	212	98.15	1,513	98.70
GG	5	1.31	1	0.60	4	1.85	20	1.30
G allele	80	10.44	32	9.58	48	11.11	294	9.59
*MTHFR* rs4845882 G>A								
GG	228	59.53	103	61.68	125	57.87	956	62.36
GA	129	33.68	54	32.34	75	34.72	512	33.40
AA	26	6.79	10	5.99	16	7.41	65	4.24
GA+AA	155	40.47	64	38.32	91	42.13	577	37.64
GG+GA	357	93.21	157	94.01	200	92.59	1,468	95.76
AA	26	6.79	10	5.99	16	7.41	65	4.24
A allele	181	23.63	74	22.16	107	24.77	642	20.94

**Table 4:** Overall and stratified analyses of *MTHFR* rs3753584 T>C, rs9651118 T>C, rs1801133 G>A, rs4846048 A>G and rs4845882 G>A polymorphisms with colorectal cancer

Genotype	Overall colorectal cancer cases (n=387)				Colon cancer (n=169)				Rectum cancer (n=218)			
	Crude OR (95%CI)	*P*	Adjusted OR^a^ (95%CI)	*P*	Crude OR (95%CI)	*P*	Adjusted OR^a^ (95%CI)	*P*	Crude OR (95%CI)	*P*	Adjusted OR^a^ (95%CI)	*P*
*MTHFR* rs3753584 T>C												
Additive model	1.20 (0.92-1.57)	0.180	1.20 (0.92-1.58)	0.178	0.92(0.61-1.38)	0.674	0.92(0.61-1.38)	0.679	**1.44(1.04-2.00)**	**0.028**	**1.44(1.04-2.00)**	**0.030**
Homozygote model	2.25 (0.89-5.68)	0.087	2.34 (0.92-5.93)	0.074	**3.51(1.23-10.01)**	**0.019**	**3.63(1.27-10.38)**	**0.016**	1.18 (0.26-5.29)	0.827	1.20 (0.27-5.38)	0.815
Dominant model	1.26 (0.97-1.63)	0.086	1.26 (0.97-1.64)	0.081	1.03(0.70-1.53)	0.867	1.04(0.70-1.53)	0.852	**1.45 (1.05-2.00)**	**0.025**	**1.44 (1.04-2.00)**	**0.026**
Recessive model	2.18(0.86-5.49)	0.100	2.26 (0.89-5.73)	0.085	**3.61(1.27-10.26)**	**0.016**	**3.74(1.31-10.64)**	**0.014**	1.09(0.25-4.88)	0.908	1.11 (0.25-4.97)	0.894
*MTHFR* rs9651118 T>C												
additive model	1.08(0.84-1.38)	0.539	1.08(0.85-1.39)	0.528	1.06(0.74-1.51)	0.762	1.06 (0.75-1.52)	0.734	1.10 (0.80-1.50)	0.556	1.10 (0.80-1.50)	0.552
homozygote model	1.34 (0.95-1.87)	0.091	1.36 (0.97-1.90)	0.077	1.56(0.98-2.47)	0.060	1.56 (0.99-2.48)	0.057	1.17 (0.75-1.82)	0.481	1.22 (0.78-1.90)	0.390
Dominant model	1.16(0.92-1.47)	0.205	1.17(0.93-1.48)	0.190	1.20(0.86-1.68)	0.290	1.21 (0.86-1.69)	0.275	1.14 (0.84-1.53)	0.397	1.15 (0.85-1.55)	0.368
Recessive model	1.29 (0.95-1.76)	0.100	1.31(0.96-1.78)	0.084	**1.53(1.01-2.31)**	**0.044**	**1.53(1.01-2.31)**	**0.044**	1.12(0.75-1.68)	0.581	1.16 (0.77-1.74)	0.471
*MTHFR* rs1801133 G>A												
additive model	0.89 (0.71-1.13)	0.331	0.89(0.70-1.12)	0.322	0.80(0.58-1.12)	0.188	0.80(0.57-1.11)	0.183	0.97(0.72-1.31)	0.834	0.97(0.72-1.30)	0.817
homozygote model	**0.56 (0.37-0.84)**	**0.006**	**0.56 (0.37-0.84)**	**0.005**	**0.47(0.25-0.87)**	**0.017**	**0.46 (0.25-0.86)**	**0.015**	0.64 (0.38-1.07)	0.089	0.64 (0.38-1.07)	0.090
Dominant model	0.83 (0.66-1.04)	0.109	0.83 (0.66-1.04)	0.106	0.74(0.54-1.02)	0.066	0.74(0.54-1.02)	0.063	0.91(0.68-1.21)	0.522	0.91(0.68-1.21)	0.512
Recessive model	**0.60 (0.40-0.89)**	**0.011**	**0.60 (0.40-0.89)**	**0.011**	**0.53 (0.29-0.96)**	**0.037**	**0.52(0.28-0.95)**	**0.035**	0.65 (0.40-1.07)	0.092	0.66 (0.40-1.08)	0.095
*MTHFR* rs4846048 A>G												
additive model	1.12(0.83-1.49)	0.466	1.11(0.83-1.49)	0.474	1.08(0.71-1.64)	0.717	1.08 (0.71-1.63)	0.735	1.14 (0.79-1.65)	0.480	1.14(0.79-1.66)	0.482
homozygote model	1.01 (0.38-2.72)	0.982	0.98 (0.36-2.63)	0.961	0.46(0.06-3.43)	0.447	0.46 (0.06-3.48)	0.454	1.45 (0.49-4.30)	0.501	1.36 (0.45-4.07)	0.583
Dominant model	1.12(0.84-1.49)	0.439	1.11(0.84-1.48)	0.457	1.05(0.69-1.58)	0.826	1.04 (0.69-1.58)	0.839	1.18 (0.82-1.68)	0.373	1.17 (0.82-1.67)	0.393
Recessive model	1.00(0.37-2.68)	1.000	0.96(0.36-2.59)	0.938	0.46(0.06-3.42)	0.445	0.46(0.06-3.45)	0.450	1.43(0.48-4.22)	0.519	1.33 (0.45-3.98)	0.609
*MTHFR* rs4845882 G>A												
additive model	1.04 (0.82-1.33)	0.741	1.05 (0.82-1.33)	0.720	0.96 (0.68-1.36)	0.832	0.97 (0.68-1.36)	0.843	1.11(0.82-1.50)	0.517	1.11(0.82-1.51)	0.508
homozygote model	**1.65 (1.03-2.66)**	**0.039**	**1.64 (1.02-2.65)**	**0.043**	1.41(0.70-2.82)	0.338	1.42 (0.71-2.85)	0.326	**1.86(1.04-3.31)**	**0.035**	1.79 (1.00-3.20)	0.050
Dominant model	1.13 (0.90-1.42)	0.308	1.13 (0.90-1.42)	0.300	1.03(0.74-1.43)	0.862	1.03(0.74-1.44)	0.845	1.21 (0.90-1.61)	0.204	1.20 (0.90-1.61)	0.211
Recessive model	**1.65(1.03-2.63)**	**0.038**	**1.63 (1.01-2.60)**	**0.044**	1.44 (0.73-2.86)	0.299	1.45 (0.73-2.88)	0.290	**1.81(1.03-3.18)**	**0.041**	1.73(0.98-3.07)	0.059

^a^Adjusted for age, sex, smoking status and alcohol use in a logistic regression model.

When compared with *MTHFR* rs1801133 GG genotype, *MTHFR* rs1801133 AA genotype was associated with a decreased risk of CRC (crude OR = 0.56, 95% CI: 0.37–0.84, *P* = 0.006). When compared with *MTHFR* rs1801133 GG/GA genotype, *MTHFR* rs1801133 AA genotype was also associated with a decreased risk of CRC (crude OR = 0.60, 95% CI: 0.40–0.89, *P* = 0.011). Adjustment for multiple factors (age, sex, smoking and drinking), the results were not essentially changed (AA vs. GG: adjusted OR = 0.56, 95% CI: 0.37–0.84, *P* = 0.005; AA vs. GG/GA: OR = 0.60, 95% CI: 0.40–0.89, *P* = 0.011; Table 4).

When compared with *MTHFR* rs4845882 GG genotype, a *MTHFR* rs4845882 AA genotype increased the risk of CRC (crude OR = 1.65, 95% CI: 1.03–2.66, *P* = 0.039). When compared with *MTHFR* rs4845882 GG/GA genotype, *MTHFR* rs4845882 AA genotype was associated with an increased risk of CRC (crude OR = 1.65, 95% CI: 1.03–2.63, *P* = 0.038). Adjustment for multiple factors (age, sex, smoking and drinking), the results were not essentially changed (AA vs. GG: adjusted OR =1.64 95% CI: 1.02–2.65, *P* = 0.043; AA vs. GG/GA: OR = 1.63, 95% CI: 1.01–2.60, *P* = 0.044; Table 4).

### Association of MTHFR rs3753584 T>C, rs9651118 T>C, rs1801133 G>A, rs4846048 A>G and rs4845882 G>A polymorphisms with CRC in a stratification group

To determine whether the effect of *MTHFR* polymorphisms was influenced by CRC region, we performed a stratified analysis. For *MTHFR* rs3753584 T>C polymorphism, we found this polymorphism was associated with an increased risk of rectum cancer (CC+TC *vs*. TT: adjusted OR = 1.44, 95% CI = 1.04–2.00, *P* = 0.026; TC *vs*. TT: adjusted OR = 1.44, 95% CI = 1.04–2.00, *P* = 0.030) and of colon cancer (CC *vs*. TT+TC: adjusted OR = 3.74, 95% CI = 1.31–10.64, *P* = 0.014; CC *vs*. TT: adjusted OR = 3.63, 95% CI = 1.27–10.38, *P* = 0.016). For *MTHFR* rs9651118 T>C polymorphism, we found *MTHFR* rs9651118 CC genotypes might be associated with an increased risk of colon cancer (CC *vs*. TT+TC: adjusted OR = 1.53, 95% CI = 1.01–2.31, *P* = 0.044). However, we found *MTHFR* rs1801133 G>A polymorphism was associated with a decreased risk of colon cancer (AA *vs*. GG+GA: adjusted OR = 0.52, 95% CI = 0.28–0.95, *P* = 0.035; AA *vs*. GG: adjusted OR = 0.46, 95% CI = 0.25–0.86, *P* = 0.015). Other comparisons are presented in Table 4.

### The power of the present study (α= 0.05)

For *MTHFR* rs3753584 T>C, the power value was 0.634 in homozygote model and 0.651 in recessive model among colon cancer group, and 0.586 in homozygote model and 0.614 in recessive model among rectum cancer group. In addition, for *MTHFR* rs9651118 T>C, the power value was 0.527 in recessive model among colon cancer group. Moreover, for *MTHFR* rs1801133 G>A, the power value was 0.815 in homozygote model and 0.748 in recessive model among overall CRC group, and 0.705 in homozygote model and 0.565 in recessive model among colon cancer group. For *MTHFR* rs4845882 G>A, the power value was 0.537 in homozygote model and 0.553 in recessive model among overall CRC group, and 0.494 in homozygote model and 0.540 in recessive model among rectum cancer group as well.

## DISCUSSION

Recently, CRC incidence and related mortality are increasing rapidly worldwide. The individual's susceptibility to CRC may be influenced by some environmental exposure and genetic factors. Recently, many case-control studies have been directed towards the association between *MTHFR* polymorphisms and CRC risk. However, the sample size of most studies was relatively small. Here, we attempt to assess the association between *MTHFR* tagging SNPs (rs3753584 T>C, rs9651118 T>C, rs1801133 G>A, rs4846048 A>G and rs4845882 G>A) and susceptibility of CRC. Our results indicated several *MTHFR* tagging polymorphisms could affect the risk of CRC.

*MTHFR* rs1801133 G>A polymorphism leads to an amino acid transformation (alanine→valine at 226 position of MTHFR protein). The role of MTHFR enzyme is remethylation of homocysteine to methionine. Polymorphisms in MTHFR gene are corrected with the deficiency of MTHFR enzyme activity. Compared with rs1801133 GG homozygote, MTHFR rs1801133 AA homozygote decreases 70% of the enzyme activity and MTHFR rs1801133 GA heterozygote loss 35% of enzymatic function. This transformation may increase the plasma homocysteine (Hcy) concentration and decreases the plasma folic acid concentration [26]. Recently, several meta-analyses indicated that *MTHFR* rs1801133 G>A polymorphism decreased the risk of CRC in Asians [27, 28]. *MTHFR* rs1801133 G>A polymorphism locates on the NH2-terminal catalytic domain. In addition, *MTHFR* rs1801133 G>A polymorphism increases the availability of 5, 10-methylenetetrahydrofolate for DNA synthesis [29, 30], which may partially explain the protective factor of CRC. In combination with this case-control study, our results evidence that G→A mutation in *MTHFR* rs1801133 increases the availability of 5, 10-methylenetetrahydrofolate for DNA synthesis; thus, this SNP may be a protective factor of CRC.

To the best of our knowledge, it was the first epidemiological study to explore the relationship of *MTHFR* rs4845882 G>A polymorphism with CRC risk. *MTHFR* rs4845882 G>A is linkage disequilibrium (LD) with rs1801131 (1298 A>C). Several meta-analyses suggested that *MTHFR* rs1801131 A>C polymorphism affected risk of CRC in Asians [31, 32]. In the present study, we found *MTHFR* rs4845882 G>A polymorphism may be associated with the development of CRC. Since *MTHFR* rs4845882 G>A and rs1801131 A>C are in strong LD (*r^2^* = 0.935, http://gvs.gs.washington.edu/GVS147/LDpairwiseR2.jsp?GET_TAYLORGRAM=1491876387794), the function of rs4845882 G>A may be affected by rs1801131 A>C.

A study reported that CC genotype of *MTHFR* rs9651118 conferred a reduced risk of breast cancer compared to TT genotype in a Chinese population [33]. Swartz *et al*. found *MTHFR* rs9651118 T>C polymorphism may be correlated with the decreased risk of lung cancer in Caucasians [34]. In our previous study, we found there was null association between *MTHFR* rs9651118 T>C polymorphism and esophageal squamous cell carcinoma in a Chinese Han population [21]. However, in this study, we found that rs9651118 CC genotype was relevant to increased risk of colon cancer. Rs9651118 T>C polymorphism is located on the intron of *MTHFR* gene. The function of this polymorphism is not well known. It was reported that *MTHFR* rs9651118 TT genotype elevated the level of Hcy compared with CC genotype [35]. In the future, more functional studies are required to identify the real biological effect of *MTHFR* rs9651118 T>C polymorphism on the etiology of CRC.

In a subgroup analysis by the region of CRC, *MTHFR* rs3753584 T>C polymorphism was associated with the risk of colon and rectum cancer. Although the function of *MTHFR* rs3753584 T>C polymorphism was not identified, a significantly increased risk of lung cancer was found for the variant allele carriers of this polymorphism, compared with individuals with wild homozygote [36]. In this study, we also found that C allele of *MTHFR* rs3753584 was probably correlated with an increased risk of colon and rectum cancer, which was consistent with the findings of those previous study.

Like all case-control studies, this study has some limitations. First, demographic variables and risk factor information only focused on age, sex, smoking and alcohol consumption. And other lifestyles were not collected, which might increase the possibility of confounding from environmental risk factors. Second, the source of non-cancer controls was hospital-based; which might not well represent the whole Chinese population. Third, in a stratified analysis by the region of CRC, the relatively small sample size may decrease the power of the results. Finally, these findings should be interpreted with very caution because the participants were only enrolled from Chinese Han population. Thus, the results may not permit extrapolation to other ethnicities.

In summary, the tagging polymorphisms in *MTHFR* gene (rs3753584 T>C, rs9651118 T>C and rs4845882 G>A) are associated with an increased risk of CRC. However, *MTHFR* rs1801133 G>A polymorphism confers a decreased risk to CRC. Our findings suggest that further validation studies are needed.

## MATERIALS AND METHODS

### Study population and patient selection

The study population consisted of 387 sporadic, consecutive CRC patients from the Department of General Surgery, Fujian Medical University Union Hospital (Fuzhou, China) between October 2014 and May 2016. Histological report confirmed the diagnosis of CRC. Non-cancer controls (n = 1,536) were randomly recruited from the Fujian Medical University Union Hospital (Fuzhou, China) and the Affiliated People's Hospital of Jiangsu University (Zhenjiang, China) between October 2014 and November 2016. The CRC patients and controls were matched for age, sex and residential area (Eastern China). Every participant was informed the aim of the present study and signed an informed consent. The protocol of the study was obtained by the institutional ethics committee of Fujian Medical University and Jiangsu University. Additionally, in this case-control study, we conformed to the principles of Declaration of Helsinki.

### Data collection

Every participant was personally questioned and answered a questionnaire regarding age, sex, the status of cigarette smoking, and alcohol consumption. Participants who drink more than thrice/week for >6 months and smoke at least one cigarette/day over 1 year were considered positive. Clinical characteristics, such as pathological stage and tumor site, were obtained from the medical records (Table 1).

### Selection of tagging SNPs

The tagging SNPs of *MTHFR* gene [30.4 Kbp spanning from 11780730 to 11811103 in chromosome 1 (upstream and downstream of the gene extending 5000 bases, respectively)] were analyzed and selected from the data of Chinese Han individuals in Beijing (CHB) via the HapMap Project (http://hapmap.ncbi.nlm.nih.gov/index.html.en) [20]. The detailed process and criterion were described previously [21]. The information of selected *MTHFR* tagging SNPs is presented in Table 2.

### DNA extraction and genotyping

Ethylenediamine tetraacetic acid-anticoagulated intravenous blood was donated by every participant. Using the Promega DNA Blood Mini Kit (Promega, Madison, USA), genomic DNA was extracted from peripheral lymphocytes by the standard experimental protocol.

A custom-by-design 48-Plex SNPscan Kit (Genesky Biotechnologies Inc., Shanghai, China), double ligation and multiplex fluorescence PCR [22], was performed to identify the genotypes of *MTHFR* rs3753584 T>C, rs9651118 T>C, rs1801133 G>A, rs4846048 A>G and rs4845882 G>A polymorphisms as described in previous studies [23, 24]. A total of 77 samples were randomly selected and were tested again for quality control. Based on 4% of duplicated samples in this study, the accordance rates were 100%.

### Statistical analysis

We used SAS statistical software, version 9.4 (SAS Institute, Cary, NC) for data analysis and a *P* < 0.05 (two–tailed) was considered to be a statistical significance. The quantitative variables were expressed as means ± standard deviation (SD). Student's t-test was harnessed to evaluate the difference of age between CRC patients and controls. Additionally, we used *χ^2^* test to examine the differences in age, sex, smoking status, alcohol consumption and the frequencies of genotype between patients with CRC and controls. The Hardy-Weinberg equilibrium (HWE) equation was used to assess whether the proportion of *MTHFR* tagging SNPs genotypes obtained was in accordance with the expected value. An online calculator (http://ihg.gsf.de/cgi-bin/hw/hwa1.pl) was harnessed to calculate the *P* value of HWE [25]. The relationship of *MTHFR* rs3753584 T>C, rs9651118 T>C, rs1801133 G>A, rs4846048 A>G and rs4845882 G>A genotypes with CRC risk was estimated by crude/adjusted odds ratios (ORs) and 95% confidence intervals (CIs). The power of the present study (α= 0.05) was evaluated by the Power and Sample Size Calculator (http://biostat.mc.vanderbilt.edu/twiki/bin/view/Main/PowerSampleSize).
